# Access to health services, food, and water during an active conflict: Evidence from Ethiopia

**DOI:** 10.1371/journal.pgph.0001015

**Published:** 2022-11-29

**Authors:** Kibrom A. Abay, Mehari Hiluf Abay, Guush Berhane, Jordan Chamberlin, Kevin Croke, Kibrom Tafere

**Affiliations:** 1 International Food Policy Research Institute, Washington DC, United States of America; 2 Department of Economics and Management, University of Florence, Firenze, Italy; 3 International Maize and Wheat Improvement Center (CIMMYT), Nairobi, Kenya; 4 Department of Global Health and Population, Harvard TH Chan School of Public Health, Boston, Massachusetts, United States of America; 5 Development Economics Group, World Bank, Washington DC, United States of America; Children’s Hospital of Eastern Ontario, University of Ottawa, CANADA

## Abstract

Civil conflict began in Ethiopia in November 2020 and has reportedly caused major disruptions in access to health services, food, and related critical services, in addition to the direct impacts of the conflict on health and well-being. However, the population-level impacts of the conflict have not yet been systematically quantified. We analyze high frequency phone surveys conducted by the World Bank, which included measures of access to basic services, to estimate the impact of the first phase of the war (November 2020 to May 2021) on households in Tigray. After controlling for sample selection, a difference-in-differences approach is used to estimate causal effects of the conflict on population access to health services, food, and water and sanitation. Inverse probability weighting is used to adjust for sample attrition. The conflict has increased the share of respondents who report that they were unable to access needed health services by 35 percentage points (95% CI: 14–55 pp) and medicine by 8 pp (95% CI:2–15 pp). It has also increased the share of households unable to purchase staple foods by 26 pp (95% CI:7–45 pp). The share of households unable to access water did not increase, although the percentage able to purchase soap declined by 17 pp (95% CI: 1–32 pp). We document significant heterogeneity across population groups, with disproportionate effects on the poor, on rural populations, on households with undernourished children, and those living in communities without health facilities. These significant disruptions in access to basic services likely underestimate the true burden of conflict in the affected population, given that the conflict has continued beyond the survey period, and that worse-affected households may have higher rates of non-response. Documented spatial and household-level heterogeneity in the impact of the conflict may help guide rapid post-conflict responses.

## Introduction and background

The large-scale civil war in Ethiopia that broke out on November 4, 2020, has triggered a deep humanitarian and health crisis. The war has led to widespread loss of life, displacement of people, damage to property, and disruptions to economic livelihoods, reversing many of the gains made in recent decades [1,2]. A recent assessment by United Nations agencies indicates that across the three conflict-affected regions (Tigray, Amhara and Afar) more than 9 million people need humanitarian food assistance, with 83 percent of people in Tigray becoming food insecure [3].

Despite such high-level assessments, the overall impact of the conflict on population-level access to critical services such as health care, food markets, and water, sanitation and hygiene (WASH) remains unknown. This is because the conflict has caused significant disruptions to telecommunication and internet services, and also because it has hampered conventional data collection efforts. This study employs a unique High-Frequency Phone Survey (HFPS) dataset to identify (near real-time) impacts of the conflict on households’ access to basic health care, food, and WASH services.

This paper aims to quantify the impacts of this ongoing large-scale conflict on households’ access to health services, food, and WASH services using Difference-in-Differences (DID) and two-way fixed effects approaches. It seeks to make several key contributions to the literature on health effects of violent conflicts. First, it uses a rigorous observational design which enables plausibly causal inferences about the impact of conflict on households’ access to critical health services and food. Second, unlike many previous studies which demonstrate long-run harms to health and well-being from early life conflict exposure [4–6], this study quantifies the impact of an ongoing conflict. This is important because representative household survey data collected during active conflicts are rare, which hinders quantification of conflict effects and planning for humanitarian intervention. Third, this paper goes beyond standard health access measures to analyze factors that are outside the health sector, but which have a central role in supporting health, such as access to food and WASH services. Fourth, this paper includes a range of heterogeneity analyses to show the differential impact on population groups within Ethiopia. Finally, this analysis adds to the emerging literature on the effects of the ongoing conflict in Ethiopia. While there are some studies which document the impact of the war on food security and livelihoods [7]; health infrastructure [8]; nutritional outcomes [9]; and anxiety and depression [10], systematic survey evidence of the conflict’s effect on access to health services, food, and water at household level has been missing.

The conflict started on November 4, 2020, when political disagreements between the Ethiopian federal government and the Tigray state turned into a full-scale war between Tigrayan forces and the federal military and its allied forces from Amhara and neighboring Eritrea. After weeks of intense fighting, federal forces and their allies captured the regional capital at the end of November 2020, and much of the region fell under their control shortly afterwards. This phase of the war was mostly confined to Tigray region. This changed when the federal and allied forces left most of Tigray in June 2021, after which the conflict spilled over into the neighboring Amhara and Afar regions. The analysis in this paper covers the first phase of the war, because the HFPS ended in May 2021, just before the withdrawal of federal forces. Over the course of the war, civilian casualties, destruction of infrastructure, large scale displacement of people, and widespread gender-based sexual violence have been documented [1,8,11–13], as has severe damage to Tigray’s health system. For example data from the Tigray Regional Health Bureau indicates that 70 percent of hospitals and over 80 percent of health centers had limited or unknown functionality due to the conflict [14]. A disturbing feature of this war has been the blockade of humanitarian aid and essential medical supplies, communication blackout and suspension of public services including banking, telephone, electricity, transport, and other basic services [1]. As a result, the complete picture of the crisis remains unknown, although previous reports have suggested significant disruptions in access to food markets, essential supplies of health and WASH services [8,11–13].

## Methods

### Data sources

#### High Frequency Phone Surveys (HFPS) data

This analysis uses household-level panel data collected by the World Bank’s High-Frequency Phone Surveys to monitor the impact of the COVID-19 pandemic on households in Ethiopia [14]. The HFPS was implemented using Computer Assisted Telephone Interviewing (CATI) and conducted every 3–4 weeks between April 2020 and May 2021. Eleven rounds of panel data were collected in total. The high frequency nature of the data allows assessment of changes in household living conditions in response to major events such as outbreak of conflict.

The HFPS sample is a subsample of households drawn from the Living Standards Measurement Study—Integrated Survey on Agriculture (LSMS-ISA), a face-to-face survey conducted in 2019. The LSMS-ISA contains a nationally representative sample of 6,770 households drawn from all regions of Ethiopia. About 79 percent of these households reported phone ownership in the 2019 survey and were subsequently used as a sampling frame for the HFPS in 2020. The sample size for the phone survey was 3,300 households, of which 3,247 were successfully interviewed in the first phone survey in April 2020. Households were re-interviewed for eleven successive rounds until May 2021 [15]. The HFPS sample declined in follow-up rounds due to non-response and attrition, especially the Tigray-based sample, due to the war itself and disruptions in telecommunication services in the region. This analysis is, therefore, restricted to the panel of 2,677 households who were interviewed before and after the first phase of the war. We discuss adjustments for differential non-response and attrition in the methods section.

The phone surveys used a similar questionnaire across rounds but followed a modular approach–some modules were dropped, and others kept or added in different rounds. In particular, questions on access to food and food markets were included in eight of the eleven rounds of the survey implemented between April 2020 and May 2021, with seven pre-war and one post-war rounds. The questions on access to health services were included in ten rounds, with six pre-war and four post-war rounds, while questions related to WASH services were limited to one pre-war and one post-war round.

#### Armed Conflict Location and Event Data (ACLED)

Using geographic coordinates of sample households at baseline, household proximity to battle locations is estimated using events recorded in the Armed Conflict Location and Event Data (ACLED), which provides event-based information for different types of conflicts, including battles and attacks against civilians. This analysis uses the battle events recorded in this data, representing clashes between conflicting forces. We construct household-level measures of exposure by counting the cumulative number of battles within 20 and 30 km radius of HFPS households’ residence at the time of each survey round.

### Definition of outcome variables

#### Access to food markets

HFPS households were asked whether they were able to buy enough staple foods (e.g., teff/injera, wheat/bread, maize and cooking oil) in the previous week. These questions capture both availability of food and ability to afford sufficient food. On one hand, the outbreak of the war has affected access to food not only by directly disrupting crop-cutting and harvesting activities but also impeding the functioning of local and inter-regional markets, especially in conflict hotspot areas. Because different staples are produced in different areas of Ethiopia, local markets may experience commodity-specific impacts of disruptions to trade. On the other hand, the war also curtailed households’ livelihood activities, contributing to likely reductions in income and the ability to afford food purchases.

#### Households’ access to health services

In the phone surveys, households were first asked if they needed any medical services (treatment or consultation) in the past 4 weeks. We define an indicator variable for health services demand which equals 1 if any member of the household needed any medical services, 0 otherwise. Households who needed health services were next asked if they were able to access the required medical services in the past 4 weeks. Therefore, conditional on the demand for health services, we define another indicator variable that takes a value of 1 if the household is unable to access the health services needed, 0 otherwise. In addition, we generate a third indicator variable that takes a value of 1 if the household is not able to buy enough medicine, 0 otherwise.

#### Households’ access to WASH

Households’ access to WASH is measured using three indicator variables, each taking value of 1 if household has sufficient access to water for drinking, sufficient access to water for washing, and sufficient access to soap for washing, 0 otherwise.

#### Estimation strategy

We employ two empirical strategies to identify the impact of the conflict on households’ access to food, health and WASH services. First, we employ a Difference-in-Differences strategy to identify the overall impacts of the war by comparing trends in households’ access to these services before and after the outbreak of the war across affected and unaffected households. Second, we use the ACLED database to construct household-level and time-varying exposure to violent conflict and estimate two-way fixed effects model. In both cases we use linear probability models (LPM); alternatively, we also present logistic regression models in the S1 Appendix. Our first approach implements the following DID specification:

$$A_{hrt}=\alpha_{h}+\alpha_{1}{Wartime}_{t}+\alpha_{2}{Tigray}_{r}\times {Wartime}_{t}+\epsilon_{hrt}$$ (1)

*A*_*hrt*_ represents access to food, health and WASH services associated with household *h* living in region *r* observed in time *t*. *α*_*h*_ captures household fixed effects. *Wartime*_*t*_ is a binary indicator of the conflict period, taking a value of 1 for periods affected by war, and 0 otherwise. The war started on November 04, 2020, but the November 2020 round interviews were completed before that date and hence appear as a pre-war round. *α*_1_ captures aggregate changes in households’ access to food, health and WASH services including in the absence of the war. *Tigray*_*r*_ represents an indicator variable for households living in Tigray region. Thus, in the first phase of the conflict, Tigray is the region affected by the conflict while the other regions in Ethiopia serve as control group. *α*_2_ captures the interaction of residence within the Tigray region and the conflict period. Under the assumptions of this DID model, this interaction term identifies the impact of the war on households’ access to food and health services. *ϵ*_*hrt*_ captures other unobserved factors that may affect households’ access to these services.

**Fig 1:**
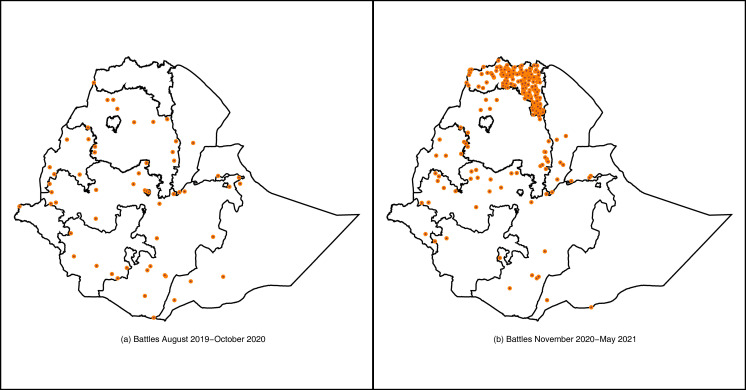
Spatial distribution of battles before and after the outbreak of the war. Source: Authors’ compilation based on ACLED data. The shapefile for Fig 1 was obtained from ArcGIS Hub. The shapefile is public domain and can be used freely.

The specification in Eq (1) assumes that all survey households from Tigray have been affected by the war and Tigray is considered as the conflict-exposed region and the rest of the country as a comparison group. This assumption is plausible, given the depth and breadth of the conflict (see Fig 1). However, some households in Tigray may not have been affected by the conflict, suggesting that the estimates in Eq (1) can only measure a parameter analogous to intention to treat (ITT) impacts; that is, the effect of living in a conflict-exposed region, rather than the effect of direct conflict exposure itself. If the share of households in Tigray who were unaffected by the armed conflict is significant, these estimates would be considerably smaller than the average treatment effect (ATE) of the war. To quantify estimates that are closer to the ATE of the war, we construct a more granular measure of exposure to violent conflict using ACLED’s battle events recorded during the survey period (between August 2019 and May 2021). We use the cumulative number of battles that took place within 20 and 30 km radius of households. This distance-based measure of exposure to battle events reduces potential misclassification of households (into affected and unaffected), while also capturing impact of conflicts realized throughout the country, including in regions bordering Tigray. While the estimates generated using these distance-based measures are still technically ITT, they are likely to be similar to the ATE of the war. Thus, we estimate the following two-way fixed effects specifications:

$$A_{hrt}=\alpha_{h}+\alpha_{m}+\varphi_{1}{Battles}_{hrt}+\omega_{hrt}$$ (2)

where *Battles*_*hrt*_ is the cumulative number of battles experienced within 20 km or 30 km radius from households’ residence. *α*_*h*_ and *α*_*m*_ stand for household and survey round fixed effects, respectively. *φ*_1_ is the main coefficient of interest, and measures the impact of an additional battle event on the outcome variables. To account for potential spatial correlation in battle experiences and the outcome variables, standard errors are clustered at district (*woreda*) level.

To account for systematic non-responses in the phone surveys, we constructed inverse probability sampling weights (used in all analyses). The sample consists of households appearing in the pre-war and at least once in the wartime phone survey rounds, implying that the weights need to be constructed considering attrition and non-responses in both phases. Households which were not observed in both pre-war and wartime rounds were considered as non-responses in the construction of sample weights. We use household and location characteristics from the 2019 face-to-face baseline survey to predict the joint probability of response in pre-war and wartime rounds (see Table F in S1 Appendix). We then construct sampling weights as the inverse of the predicted probability of responses in both pre-war and post-war-onset phone surveys. We note that applying the weights markedly reduces the differences between the unweighted means in the observable characteristics of baseline (in person) and phone surveys (Table G in S1 Appendix). In particular, Table G in S1 Appendix shows that applying the sampling weights renders observable characteristics of the phone survey sample largely comparable to the full sample. The statistical software used for the analysis in this paper is Stata 17.0.

### Ethics

The Institutional Review Board (IRB) of the Harvard T.H. Chan School of Public Health determined that this study did not qualify as human subjects research (Protocol number IRB22-0341), as the study uses only publicly available, anonymized secondary data.

## Results

### Descriptive results

Fig 1 presents the distribution of violent conflict events, in all regions of Ethiopia before (August 2019-October 2020) and after the outbreak of the war (November 2020-May 2021) based on conflict event records in ACLED data. Battle events were relatively rare and more evenly distributed across regions before November 2020, except in Tigray where there were no battles (Panel (a)). During the November 2020—May 2021 period, there was a dramatic spike in battle events, the vast majority of which taking place in Tigray, with little change elsewhere (Panel (b)).

Table 1 shows pooled summary statistics of the study outcomes. 24 percent of households report being unable to purchase some types of food. When disaggregated by item, about 11 percent of households were not able to purchase *teff* (panel (a)), the most important staple food in Ethiopia. About 30 percent of households had some need for health services in the week prior to the survey, of which 11 and 6 percent of households were unable to access the health services and not able to purchase medicine, respectively (panel (b)). About 24 percent of households lacked sufficient drinking water, while 7 percent lacked washing water and 11 percent lacked access to soap (panel (c)).

**Table 1:** Summary of households’ access to health services, food markets, and WASH, all survey rounds.

	Observations	Mean	95% conf. interval
(a) Access to food			
Not able to buy enough food	19,495	0.24	[0.23,0.24]
Not able to buy teff/injera	19,495	0.11	[0.11,0.12]
Not able to buy wheat	19,495	0.10	[0.09,0.10]
Not able to buy maize	19,495	0.05	[0.05,0.05]
Not able to buy edible oil	19,495	0.15	[0.15,0.16]
(b) Access to health services			
Demand for health services (treatment or consultation)	26,259	0.30	[0.29,0.30]
Unable to access to health services (treatment or consultation)	7,708	0.11	[0.10,0.12]
Unable to buy enough medicine	19,495	0.06	[0.05,0.06]
(c) Access to WASH services			
Access to sufficient water for drinking	4,563	0.76	[0.75,0.77]
Access to sufficient water for washing	4,563	0.93	[0.93,0.94]
Access to sufficient soap for washing	4,563	0.89	[0.88,0.90]

Notes: Sample size for each outcome by survey round are provided in Table A in S1 Appendix.

### Difference-in-differences results

**Fig 2:**
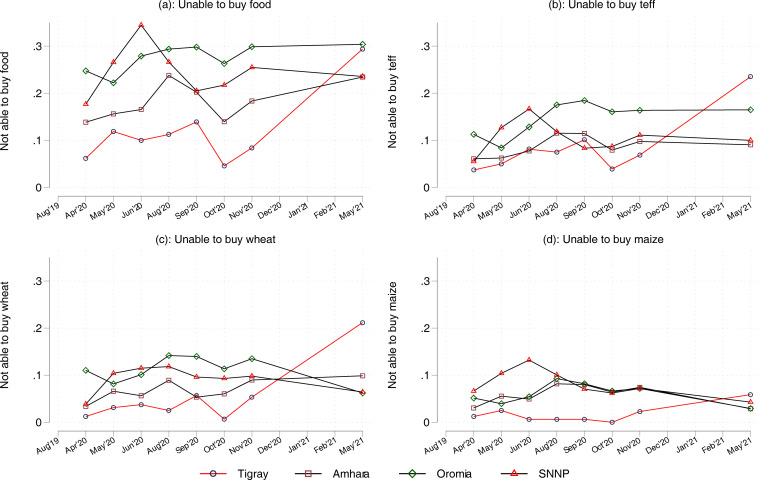
Access to food markets.

Fig 2 shows trends in households’ ability to purchase staple foods from the market. Panel (a) shows trends in access to food items in the four major highland regions of Ethiopia. Before the war, the share of households reporting inability to buy enough food was lowest in Tigray. This figure increased from 5 percent in October 2020 (just prior to the start of the war) to 29 percent in May 2021. The temporal trends in households’ inability to buy teff, wheat and maize is shown in panels (b)-(d). In all three panels, access to food markets remained relatively stable over time for Amhara, Oromia and SNNP regions, while in Tigray, the share of households that reported not being able to purchase teff increased from 5 to 23 percent between the last pre-conflict survey round (October 2020) and the final survey round (May 2021). The corresponding value for wheat increased from 2 to 21 percent.

Table 2 provides estimation results for regression models of the factors associated with food access outcomes. The interaction term between the indicator variable for Tigray and wartime indicator variable captures differential trends in households’ access to food markets between Tigray and the rest of Ethiopia; this is the quantity of interest in our model. The first column provides results associated with households’ ability to buy sufficient foods of any type, while the remaining columns show results for specific foods. Households reporting inability to buy enough staple foods increased by 5 percentage points in those regions outside Tigray in the wartime period but by an additional 26 percentage points in Tigray region. The disaggregated results indicate larger disruptions to households’ ability to access teff, an inter-regionally traded staple, and wheat. By contrast, impact estimates are smaller for maize and statistically insignificant for oil.

**Table 2:** The impact of violent conflict on households’ access to food markets; difference-in-difference models.

	(1)	(2)	(3)	(4)	(5)
	Unable to buy enough food	Unable to buy teff	Unable to buy wheat	Unable to buy maize	Unable to buy oil
Wartime period	0.05**	-0.03	0.04**	0.00	0.06***
	[0.01,0.09]	[-0.06,0.01]	[0.00,0.08]	[-0.02,0.02]	[0.02,0.10]
Tigray × Wartime period	0.26***	0.30***	0.24**	0.05*	0.14
	[0.07,0.45]	[0.11,0.49]	[0.04,0.43]	[-0.01,0.10]	[-0.06,0.34]
Household fixed effect	Yes	Yes	Yes	Yes	Yes
R-squared	0.01	0.01	0.01	0.00	0.01
Dependent variable mean (pre-war)	0.16	0.08	0.06	0.04	0.10
Observations	19495	19495	19495	19495	19495

Notes: The regression equation for this analysis is given in Eq (1). The outcome variables in this table come from a series of questions eliciting whether a household was unable to buy enough of the above staple foods in the last 7 days. The first column provides results associated with households’ ability to buy enough foods while the remaining columns provide impacts on specific types of staples. All estimations use sampling weights to capture systematic non-response and attrition in phone surveys. 95% confidence interval with clustered standard errors at district (woreda) level, are given in parentheses.

* *p* < 0.10

** *p* < 0.05

*** *p* < 0.01.

**Fig 3:**
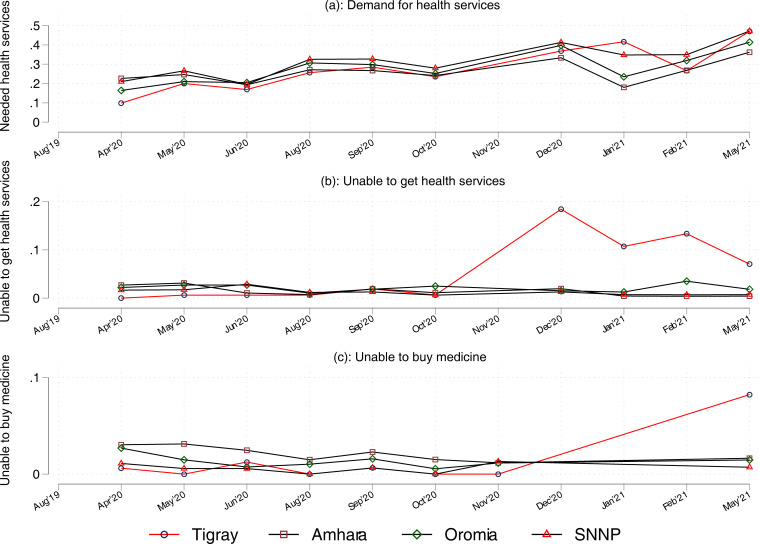
Households’ access to health services.

Fig 3 provides temporally and spatially disaggregated trends in households’ access to health services. Panel (a) shows that the patterns of demand for health services in Tigray did not change appreciably after the start of the war in November 2020. However, the share of households who were unable to access health services (panels (b)) or buy medicines (panel (c)) had sharply increased.

Columns 1–3 of Table 3 provide estimates of the impacts of the conflict on households’ access to health services using the DID regression framework. The first column shows the impact of the conflict on demand for health services, while the remaining two columns show the impacts on access to health services and medicine among households who reported need for health services. Among this group, the war reduced access by 35 percentage points, while households’ ability to buy medicine dropped by 8 percentage points.

**Table 3:** The impact of violent conflict on access to health and WASH services.

	Health services			WASH services		
	Demand for health service	Unable to get health service	Unable to buy medicine	Access to drinking water	Access to washing water	Access to Soap
Wartime period	0.09***	-0.05***	-0.01	-0.03	-0.04***	0.02
	[0.07,0.11]	[-0.08,-0.03]	[-0.03,0.01]	[-0.07,0.01]	[-0.07,-0.02]	[-0.02,0.05]
Tigray×Wartime period	-0.00	0.35***	0.08**	0.03	-0.10	-0.17**
	[-0.09,0.09]	[0.14,0.55]	[0.02,0.15]	[-0.19,0.25]	[-0.35,0.16]	[-0.32,-0.01]
Household fixed effect	Yes	Yes	Yes	Yes	Yes	Yes
R-squared	0.01	0.03	0.00	0.00	0.03	0.01
Dependent variable mean (pre-war)	0.27	0.09	0.02	0.78	0.96	0.92
Observations	26259	7708	19495	4563	4563	4563

Notes: The regression equation for this analysis is given in Eq (1). The health outcome variable in the first column is demand for health/medical service in the last four weeks. The second column measures whether households were able to access medical services provided they needed them. The outcome variable in the third column is an indicator variable assuming a value of 1 if households were unable to buy medicine in the last 7 days. The WASH outcome variables in columns 4–6 are indicator variables that take a value 1 if the household had access to enough drinking water, access to washing water, and access to enough washing soap, respectively. All estimations use sampling weights to capture systematic non-response and attrition in phone surveys. 95% confidence interval with clustered standard errors at district (woreda) level, are given in parentheses.

* *p* < 0.10

** *p* < 0.05

*** *p* < 0.01.

Columns 4–6 of Table 3 show regression estimates of impacts of the conflict on households’ access to WASH services: access to enough drinking water, access to enough washing water, and access to enough washing soap. The results in columns 4–5 indicate that war caused no significant change in access to drinking or washing water. However, households’ ability to access enough soap decreased by 16.6 percentage points.

### Heterogeneity analyses

In Table 4, the sample is divided into rural and urban areas, poor and non-poor households and households with and without undernourished children under age 5, and the empirical specification in Eq (1) is re-estimated. Poor households and households with undernourished children are identified using the (baseline) face-to-face LSMS survey in August 2019. Households in the bottom three quintiles of the welfare distribution (consumption expenditure) are defined as poor. Households with undernourished children are those with any child under the age of 5 whose height or length and body weight qualified as wasted, stunted, or underweight (2 standard deviations below mean) using WHO reference populations. Formal tests for the heterogeneity of treatment effects (p-value) are provided for each panel. Rural households (Panel A) and poorer households (Panel B) experienced disproportionally higher reductions in access and ability to buy staple foods. For example, poorer households experienced 38 percentage point reduction in their ability to buy teff, almost twice the effect on non-poor households. Households including children with pre-war nutritional deficits also suffered more because of the conflict relative to households without undernourished children.

**Table 4:** The impact of violent conflict on households’ access to food markets: Heterogeneity across rural and urban areas; across poor and non-poor households, and across households with and without nutritional deficits.

	Unable to buy teff		Unable to buy wheat		Unable to buy maize		Unable to buy oil	
Panel A: Rural versus urban households								
	Rural	Urban	Rural	Urban	Rural	Urban	Rural	Urban
Wartime period	-0.05	-0.01	0.08**	0.01	0.02	-0.01	0.02	0.09***
	[-0.13,0.03]	[-0.03,0.01]	[0.00,0.17]	[-0.02,0.03]	[-0.03,0.07]	[-0.02,0.00]	[-0.07,0.10]	[0.05,0.13]
Tigray × Wartime period	0.84***	0.14***	0.72***	0.12**	0.13	0.03**	0.67***	-0.03
	[0.73,0.95]	[0.08,0.21]	[0.61,0.82]	[0.02,0.22]	[-0.13,0.38]	[0.00,0.06]	[0.37,0.96]	[-0.09,0.04]
Interaction equality test (p-values)	0.00		0.00		0.08		0.00	
Observations	5228	14267	5228	14267	5228	14267	5228	14267
Panel B: Poor versus non-poor households								
	Poor	Non-poor	Poor	Non-poor	Poor	Non-poor	Poor	Non-poor
Wartime period	-0.01	-0.05**	0.02	0.07**	0.00	0.00	0.04*	0.09***
	[-0.05,0.03]	[-0.10,-0.01]	[-0.01,0.06]	[0.01,0.13]	[-0.03,0.03]	[-0.03,0.03]	[-0.00,0.09]	[0.04,0.14]
Tigray × Wartime period	0.38***	0.20**	0.32**	0.12	0.04	0.06	0.26*	-0.04
	[0.12,0.64]	[0.04,0.37]	[0.04,0.61]	[-0.08,0.32]	[-0.04,0.12]	[-0.02,0.13]	[-0.04,0.57]	[-0.12,0.04]
Interaction equality test (p-values)	0.00		0.00		0.65		0.00	
Observations	11414	8081	11414	8081	11414	8081	11414	8081
Panel C: Households with versus without undernourished children								
	With nutritional deficit	Without nutritional deficit	With nutritional deficit	Without nutritional deficit	With nutritional deficit	Without nutritional deficit	With nutritional deficit	Without nutritional deficit
Wartime period	-0.02	-0.03*	0.09**	0.03	0.04	-0.01	0.00	0.07***
	[-0.09,0.06]	[-0.06,0.00]	[0.00,0.17]	[-0.01,0.07]	[-0.02,0.11]	[-0.03,0.01]	[-0.08,0.08]	[0.03,0.11]
Tigray × Wartime period	0.51**	0.23***	0.44**	0.16***	0.01	0.06*	0.49**	0.02
	[0.11,0.91]	[0.13,0.32]	[0.06,0.81]	[0.04,0.28]	[-0.12,0.14]	[-0.01,0.12]	[0.09,0.89]	[-0.07,0.11]
Interaction equality test (p-values)	0.00		0.00		0.35		0.00	
Observations	2690	16805	2690	16805	2690	16805	2690	16805

Notes: The outcome variables in this table come from a series of questions eliciting whether a household was unable to buy enough of the above staple foods in the last 7 days. Panel A provides disaggregated results for rural and urban households while Panel B reports results for poor and non-poor households. Panel C reports results for households with and without underlying nutritional deficits. All estimations control for household fixed effects. All estimations use sampling weights to capture systematic non-response and attrition in phone surveys. 95% confidence interval with clustered standard errors at district (woreda) level, are given in parentheses.

* *p* < 0.10

** *p* < 0.05

*** *p* < 0.01.

We also estimate the impact of the conflict on households’ access to health services, by splitting the sample into communities with and without access to health services before the outbreak of the conflict (Table 5, Panel A). Communities without a health center experienced a 60 percentage point reduction in access to health services, almost three times the effect on those communities with health services pre-war (Table 5, Panel A).

**Table 5:** The Impact of violent conflict on households’ access to health and WASH services: Impact heterogeneity across communities with and without health facilities, and across rural and urban areas.

	Panel A: Health services across communities with and without health facilities					
	Demand for health service		Unable to get health service		Unable to buy medicine	
	Community has no health center	Community has health center	Community has no health center	Community has health center	Community has no health center	Community has health center
Wartime period	0.12***	0.07***	-0.07**	-0.04***	-0.03	-0.01
	[0.08,0.16]	[0.05,0.09]	[-0.13,-0.02]	[-0.07,-0.02]	[-0.08,0.02]	[-0.03,0.01]
Tigray ×Wartime period	0.08	-0.04	0.60***	0.23**	0.11	0.08**
	[-0.05,0.21]	[-0.14,0.07]	[0.36,0.85]	[0.02,0.44]	[-0.03,0.25]	[0.01,0.15]
Interaction equality test (p-values)	0.01		0.00		0.93	
Observations	7055	18742	2152	5465	2939	16213
	Panel B: WASH services across rural and urban areas					
	Access to drinking water		Access to washing water		Access to soap	
	Rural	Urban	Rural	Urban	Rural	Urban
Wartime period	0.01	-0.06***	-0.07***	-0.02	-0.00	0.03***
	[-0.05,0.07]	[-0.11,-0.02]	[-0.12,-0.03]	[-0.06,0.01]	[-0.08,0.07]	[0.02,0.05]
Tigray ×Wartime period	0.17	-0.08	0.08	-0.23**	-0.20	-0.14***
	[-0.19,0.52]	[-0.25,0.08]	[-0.38,0.54]	[-0.44,-0.03]	[-0.52,0.12]	[-0.24,-0.04]
Interaction equality test (p-values)	0.00		0.00		0.38	
Observations	1241	3322	1241	3322	1241	3322

Notes: The outcome variable in the first and second columns of Panel A is demand for health/medical service in the last four weeks. The third and fourth columns in Panel A measure whether households were able to access medical services provided they needed them. The outcome variable in the last two columns of Panel A is an indicator variable assuming a value of 1 if households were unable to buy medicine in the last 7 days. The outcome variable in the first two columns of Panel B characterizes access to enough drinking water while the third and fourth columns provide results on access to washing water. The dependent variable in the final two columns of Panel B is access to enough washing soap. All estimations control for household fixed effects. All estimations use sampling weights to capture systematic non-response and attrition in phone surveys. 95% confidence interval with clustered standard errors at district (woreda) level, are given in parentheses.

* *p* < 0.10

** *p* < 0.05

*** *p* < 0.01.

Finally, we explore potential heterogeneities in impacts on households’ access to WASH services by rural and urban areas. The results shown in Table 5 (Panel B) indicate that the impact of the war on WASH services was higher in urban areas. While we find no effect on access to drinking water, households living in urban areas experienced a 23.5 percentage points reduction in access to washing water and 13.8 percentage point reduction in access to soap.

### Fixed effect results: Proximity to large scale conflict events (battles)

As a counterpart to the regional DID models, regression models of the impacts of proximity to battles show qualitatively similar results. Table B in S1 Appendix shows the estimated impacts of the number of battles within 20 and 30km on food access outcomes. For each additional battle that takes place within 20 km in the months preceding the survey, the likelihood of being unable to buy enough food increased by 0.6 percentage points. These disruptions were most pronounced for teff and wheat, with smaller impacts on maize and oil. In Table C in S1 Appendix, we present results from similar models in which the dependent variables are measures of access to health services. Each battle within 20km is associated with a 0.8 percentage point increase in the likelihood of not being able to access health services and a 0.3 percentage point increase in the likelihood of being unable to access medicine.

Because our outcome variables are binary in nature and as a sensitivity analysis, we also run all our estimations using standard and fixed effects logit regressions. These results show qualitatively similar findings (Tables D and E in S1 Appendix).

## Discussion

This study examines the impacts of an ongoing large-scale conflict in the Tigray region of Ethiopia on households’ access to health services, food, and WASH services. Using a high frequency phone survey data conducted on a panel of 2,677 households and a difference-in-differences methodology, the central findings are that conflict exposure has large, statistically significant negative impacts on access to health services and food. These impacts were more pronounced on the poorer than non-poor, on households with undernourished children, and on rural compared to urban households. Although not unexpected, the magnitude of the disruptions we document in terms of access to food, basic health services and sanitation items are strikingly high given these were measured immediately after the first phase of the conflict, suggesting the likelihood of deeper subsequent impacts with the continued blockade of access to these services for months afterwards.

These results echo previous evidence on the dramatic negative consequences of armed conflicts on public health services and public health delivery [16]. While there is a large literature documenting the multiple harmful impacts of war, the findings of this study document the magnitude of the harms *ex durante* (i.e. while the conflict is still ongoing), their localized nature, and the additional harms suffered by already disadvantaged groups. The large impacts recorded–including the dramatic decrease in access to health care among those in need of treatment–likely reflects both restrictions on mobility and harm to livelihoods, as well as the destruction of public health services and facilities documented recently [8,9,17,18].

Similarly, the magnitude of the disruption to food systems is striking. The ability to acquire staple foods is dramatically affected, with disruptions occurring through both price effects (likely reflecting diminished supply, due to supply chain disruptions) and reductions in household’s ability to afford food (because of reductions in income). These findings are consistent with previous studies showing increased malnutrition in Tigray after the onset of the conflict [9].

The pronounced heterogeneity in impacts across geography, income, nutritional status, and baseline access to services is also consistent with previous literature. The fact that rural households have experienced larger disruptions in access to food and health services reflects the more tenuous nature of rural markets and services, relative to those in urban areas. Given the reported road blockages and disruptions in public transport, it is not surprising that rural markets and public health services are relatively more affected. In addition, because rural households in Ethiopia are poorer on average than urban households, food price increases are likely more problematic in these areas. These varying impacts of the conflict across different value chains is important and consistent with other recent studies [19]. In contrast, however, our finding that urban households have suffered greater disruptions to sanitation services accords with their greater reliance on piped water infrastructure, which has been extensively damaged in the conflict.

Recent reviews have highlighted major data gaps with respect to health service availability, utilization, and health outcomes in conflict settings [20,21]. These reviews have called for increased use of various data collection strategies to fill these gaps, including the Health Resources Availability Mapping System (HeRAMS) [22], surveys in camps for displaced persons, improved administrative data, and improved coordination of existing and new data tools [23], to quantify gaps in access to health services. This paper demonstrates the potential for mobile phone surveys to also play an important role in filling gaps, especially when a pre-conflict baseline survey has been conducted.

As with many studies relying on phone surveys, our analysis is constrained by limitations associated with non-random selection and non-response. While the pre-conflict LSMS-ISA sample in Ethiopia is nationally representative, the follow-up phone surveys faced attrition, in part due to the war’s effects. While we employ the inverse probability sampling weights, our weighting may not fully capture systematic differences between responding and non-responding households. Under the seemingly plausible assumption that more conflict-affected households are less likely to be able to respond to phone surveys, these results represent a lower bound of the actual impacts of the conflict on the outcomes that we study. Furthermore, although the difference-in-difference methodology adjusts for time-invariant and pre-exposure differences in the outcome of interest between exposed and non-exposed communities, residual time-varying confounding factors, such as variables correlated with conflict exposure and residence in Tigray region, remain possible threats to a causal interpretation of these results. Another limitation is that household exposure to conflict could be misclassified due to population movements. However, this misclassification of exposure would typically result in attenuation bias, suggesting that the reported findings may underestimate the harms of the ongoing conflict. In addition, while our analysis captures service disruptions from the perspective of system users; future analysis could incorporate information on disruptions to health facility functioning. A further limitation is that the LSMS-ISA HFPS only continued until May 2021. As a result, we can only examine the impact of the first phase of the conflict. Further research to understand the ongoing dynamics of the conflict after this initial phase are warranted. The results emphasize that policy actions to mitigate the harmful effects on populations are urgently needed.

## Supporting information

S1 Appendix Tables A-G. Table A: Number of households interviewed for main outcome variables. Table B: Local conflict events and access to food markets. Table C: Local conflict events and access to health services. Table D: The impact of violent conflict on households’ access to food markets; logit and fixed effects logit models. Table E: The impact of violent conflict on access to health and WASH services: logit models. Table F: Modeling the probability of response in both pre-and post-war-onset phone surveys. Table G: Descriptive statistics of the sample, by sample weights.
(DOCX)
