# Supplementary material for: Access to health services, food, and water during an active conflict: Evidence from Ethiopia
Source: PLOS Glob Public Health. 2022 Nov 29;2(11):e0001015. doi: 10.1371/journal.pgph.0001015 (PMC10021679; doi:10.1371/journal.pgph.0001015)
Supplement: S1 Appendix — Table A: Number of households interviewed for main outcome variables. Table B: Local conflict events and access to food markets. Table C: Local conflict events and access to health services. Table D: The impact of violent conflict on households’ access to food markets; logit and fixed effects logit models. Table E: The impact of violent conflict on access to health and WASH services: logit models. Table F: Modeling the probability of response in both pre-and post-war-onset phone surveys. Table G: Descriptive statistics of the sample, by sample weights. (DOCX) [file pgph.0001015.s001.docx]

**S1 Appendix**

**Tables**

Table A: Number of households interviewed for main outcome variables

|  | 20-Apr | 20-May | 20-Jun | 20-Aug | 20-Sep | 20-Oct | 20-Nov | 20-Dec | 21-Jan | 21-Feb | 21-May | Total |
| --- | --- | --- | --- | --- | --- | --- | --- | --- | --- | --- | --- | --- |
| Not able to buy enough food | 2,672 | 2,605 | 2,592 | 2,512 | 2,435 | 2,420 | 2,299 | 0 | 0 | 0 | 1,960 | 19,495 |
| Not able to buy teff/injera | 2,672 | 2,605 | 2,592 | 2,512 | 2,435 | 2,420 | 2,299 | 0 | 0 | 0 | 1,960 | 19,495 |
| Not able to buy wheat | 2,672 | 2,605 | 2,592 | 2,512 | 2,435 | 2,420 | 2,299 | 0 | 0 | 0 | 1,960 | 19,495 |
| Not able to buy maize | 2,672 | 2,605 | 2,592 | 2,512 | 2,435 | 2,420 | 2,299 | 0 | 0 | 0 | 1,960 | 19,495 |
| Not able to buy edible oil | 2,672 | 2,605 | 2,592 | 2,512 | 2,435 | 2,420 | 2,299 | 0 | 0 | 0 | 1,960 | 19,495 |
| Needed medical treatment | 2,672 | 2,605 | 2,592 | 2,512 | 2,435 | 2,420 | 0 | 2,194 | 2,051 | 2,146 | 1,960 | 26,259 |
| Unable to access needed medical treatment | 492 | 635 | 517 | 758 | 721 | 658 | 0 | 802 | 630 | 644 | 743 | 7,708 |
| Unable to buy enough medicine | 2,672 | 2,605 | 2,592 | 2,512 | 2,435 | 2,420 | 2,299 | 0 | 0 | 0 | 1,960 | 19,495 |
| Had enough drinking water | 0 | 0 | 0 | 2,512 | 0 | 0 | 0 | 0 | 2,051 | 0 | 0 | 4,563 |
| Had enough handwashing water | 0 | 0 | 0 | 2,512 | 0 | 0 | 0 | 0 | 2,051 | 0 | 0 | 4,563 |
| Had enough handwashing soap | 0 | 0 | 0 | 2,512 | 0 | 0 | 0 | 0 | 2,051 | 0 | 0 | 4,563 |

Notes: This table presents the number of observations for the main outcome variables of the paper by survey round.

Table B: Local battles and access to food markets

|  | Unable to buy enough food | Unable to buy teff | Unable to buy wheat | Unable to buy maize | Unable to buy oil |
| --- | --- | --- | --- | --- | --- |
| Panel A: Using battles within 20 km | | | | | |
| Battles within 20km | 0.06^**^ | 0.07^***^ | 0.05^*^ | 0.01 | 0.02 |
|  | [0.01,0.10] | [0.03,0.12] | [-0.00,0.10] | [-0.00,0.02] | [-0.02,0.07] |
| Household fixed effect | Yes | Yes | Yes | Yes | Yes |
| R-squared | 0.01 | 0.02 | 0.02 | 0.00 | 0.01 |
| Mean dep. var (pre-war) | 0.16 | 0.08 | 0.06 | 0.04 | 0.10 |
| No. observations | 19293 | 19293 | 19293 | 19293 | 19293 |
| Panel A: Using battles within 30 km | | | | | |
| Battles within 30km | 0.04^**^ | 0.06^***^ | 0.04^**^ | 0.01^**^ | 0.02 |
|  | [0.01,0.08] | [0.02,0.09] | [0.00,0.08] | [0.00,0.02] | [-0.02,0.05] |
| Household fixed effect | Yes | Yes | Yes | Yes | Yes |
| R-squared | 0.01 | 0.02 | 0.02 | 0.00 | 0.01 |
| Mean dep. var (pre-war) | 0.16 | 0.08 | 0.06 | 0.04 | 0.10 |
| No. observations | 19293 | 19293 | 19293 | 19293 | 19293 |

Notes: The outcome variables in this table come from a series of questions eliciting whether a household was unable to buy enough of the above staple foods in the last 7 days. The number of battles has been rescaled to achieve appropriate coefficients in 2 decimal points; and these coefficients measure impact per 10 battles. All estimations use sampling weights to capture systematic non-response and attrition in phone surveys. 95% confidence interval with clustered standard errors at district (woreda) level, are given in parentheses. ^*^ *p* < 0.10, ^**^ *p* < 0.05, ^***^ *p* < 0.01.

Table C: Local battles and access to health services

|  | (1) | (2) | (3) | (4) | (5) | (6) |
| --- | --- | --- | --- | --- | --- | --- |
|  | Demand for health services | Demand for health services | Unable to get health service | Unable to get health service | Unable to buy medicine | Unable to buy medicine |
| Battles within 20km | -0.002 |  | 0.08^**^ |  | 0.03^***^ |  |
|  | [-0.03,0.03] |  | [0.01,0.14] |  | [0.01,0.05] |  |
| Battles within 30km |  | -0.005 |  | 0.06^**^ |  | 0.02^***^ |
|  |  | [-0.03,0.02] |  | [0.00,0.12] |  | [0.01,0.03] |
| Household fixed effects | Yes | Yes | Yes | Yes | Yes | Yes |
| R-squared | 0.05 | 0.05 | 0.05 | 0.06 | 0.04 | 0.04 |
| Mean dep. var (pre-war) | 0.27 | 0.27 | 0.09 | 0.09 | 0.02 | 0.02 |
| No. observations | 25972 | 25972 | 7587 | 7587 | 19293 | 19293 |

Notes: The outcome variable in the first and second columns is demand for health/medical service in the last four weeks. The third and fourth columns measure whether households were able to access medical services provided they needed them. The outcome variable in the last two columns is an indicator variable assuming a value of 1 if households were unable to buy medicine in the last 7 days. The number of battles has been rescaled to achieve appropriate coefficients in 2 decimal points; and these coefficients measure impact per 10 battles. All estimations use sampling weights to capture systematic non-response and attrition in phone surveys. 95% confidence interval with clustered standard errors at district (woreda) level, are given in parentheses. ^*^ *p* < 0.10, ^**^ *p* < 0.05, ^***^ *p* < 0.01.

Table D: The impact of violent conflict on households’ access to food markets; logit and fixed effects logit models

|  | (1) | (2) | (3) | (4) | (5) |
| --- | --- | --- | --- | --- | --- |
|  | Unable to buy enough food | Unable to buy teff | Unable to buy wheat | Unable to buy maize | Unable to buy oil |
| Panel A: Logit model coefficients | | | | | |
| Wartime period | 0.42^***^ | -0.02 | 0.13 | -0.29^**^ | 0.62^***^ |
|  | [0.31,0.54] | [-0.19,0.15] | [-0.05,0.31] | [-0.57,-0.02] | [0.50,0.75] |
| Tigray × Wartime period | 0.95^***^ | 1.52^***^ | 1.99^***^ | 2.01^***^ | 0.63^*^ |
|  | [0.43,1.47] | [0.93,2.10] | [1.34,2.64] | [0.91,3.12] | [-0.08,1.35] |
| Household fixed effect | No | No | No | No | No |
| No. observations | 19719 | 19719 | 19719 | 19719 | 19719 |
| Panel B: fixed effects logit model coefficients | | | | | |
| Wartime period | 0.74^***^ | 0.03 | 0.26^**^ | -0.29^*^ | 0.97^***^ |
|  | [0.59,0.89] | [-0.17,0.23] | [0.05,0.47] | [-0.60,0.02] | [0.81,1.12] |
| Tigray × Wartime period | 1.35^***^ | 2.36^***^ | 2.29^***^ | 3.66^***^ | 1.06^**^ |
|  | [0.67,2.03] | [1.49,3.24] | [1.46,3.12] | [1.47,5.86] | [0.11,2.01] |
| Household fixed effect | Yes | Yes | Yes | Yes | Yes |
| No. observations | 9364 | 5808 | 4997 | 3097 | 7510 |

Notes: These results are coefficients from fixed effects logit regressions. Panel A provides results based on standard logit while the results in Panel B come from a fixed effects logit regression. The outcome variables in this table come from a series of questions eliciting whether a household was unable to buy enough of the above staple foods in the last 7 days. The first column provides results associated with households’ ability to buy enough foods while the remaining columns provide impacts on specific types of staple foods. The sample sizes in Panel B are smaller compared to Panel A and the main results in Table 2 because the fixed effects logit models rely on those observations whose outcome variables vary across time. 95% confidence interval with clustered standard errors at district (woreda) level, are given in parentheses. ^*^ *p* < 0.10, ^**^ *p* < 0.05, ^***^ *p* < 0.01.

Table E: The impact of violent conflict on access to health and WASH services: logit models

|  | Health services | | | WASH services | | |
| --- | --- | --- | --- | --- | --- | --- |
|  | Demand for  health service | Unable to get  health service | Unable to buy medicine | Access to  drinking water | Access to  washing water | Access to  Soap |
|  | Panel A: Logit model coefficients | | | | | |
| Wartime period | 0.29^***^ | -0.86^***^ | -0.18 | -0.26^***^ | -0.56^***^ | 0.45^***^ |
|  | [0.24,0.35] | [-1.08,-0.64] | [-0.51,0.15] | [-0.40,-0.12] | [-0.82,-0.30] | [0.21,0.69] |
| Tigray×Wartime period | 0.34^**^ | 2.58^***^ | 3.37^***^ | -0.50^*^ | -1.40^***^ | -2.11^***^ |
|  | [0.08,0.59] | [1.98,3.18] | [2.08,4.66] | [-1.08,0.08] | [-2.23,-0.58] | [-3.33,-0.90] |
| Household fixed effect | No | No | No | No | No | No |
| No. observations | 26570 | 7810 | 19719 | 4619 | 4619 | 4619 |
|  | Panel B: Fixed effects logit model coefficients | | | | | |
| Wartime period | 0.39^***^ | -0.93^***^ | -0.004 | -0.42^***^ | -0.69^***^ | 0.56^***^ |
|  | [0.32,0.45] | [-1.22,-0.65] | [-0.38,0.37] | [-0.60,-0.24] | [-0.98,-0.39] | [0.25,0.88] |
| Tigray×Wartime period | 0.25^*^ | 3.04^***^ | 16.50 | -0.33 | -1.19^**^ | -16.62 |
|  | [-0.03,0.53] | [2.20,3.89] | [-1063.62,1096.62] | [-1.14,0.48] | [-2.28,-0.09] | [-1920.56,1887.31] |
| Household fixed effect | Yes | Yes | Yes | Yes | Yes | Yes |
| No. observations | 22374 | 1754 | 1778 | 1054 | 448 | 362 |

Notes: These results are coefficients from standard logit regressions. The health outcome variable in the first column is demand for health/medical service in the last four weeks. The second column measures whether households were able to access medical services provided they needed them. The outcome variable in the third column is an indicator variable assuming a value of 1 if households were unable to buy medicine in the last 7 days. The WASH outcome variables in columns 4–6 are indicator variables that take a value 1 if the household had access to enough drinking water, access to washing water, and access to enough washing soap, respectively. 95% confidence interval with clustered standard errors at district (woreda) level, are given in parentheses. . ^*^ *p* < 0.10, ^**^ *p* < 0.05, ^***^ *p* < 0.01.

Table F: Modeling the probability of response in both pre-and post-war-onset phone surveys

| **Explanatory variables** | **Coefficients** |
| --- | --- |
| Household head age in years | 0.004 |
|  | (0.002) |
| Household head is female | 0.055 |
|  | (0.069) |
| Education of household head: Upper primary | 0.026 |
|  | (0.089) |
| Education of household head: Secondary or higher | 0.318^***^ |
|  | (0.088) |
| Household size | -0.012 |
|  | (0.021) |
| Adult household members size | 0.047 |
|  | (0.034) |
| Household head engaged in agriculture | -0.020 |
|  | (0.161) |
| Household head engaged in wage earning | 0.020 |
|  | (0.148) |
| Household head engaged in non-farm business | -0.105 |
|  | (0.142) |
| Asset quintile: Second | 0.187^*^ |
|  | (0.104) |
| Asset quintile: Third | 0.474^***^ |
|  | (0.115) |
| Asset quintile: Fourth | 0.833^***^ |
|  | (0.127) |
| Asset quintile: Fifth | 0.876^***^ |
|  | (0.149) |
| Household located in rural area | -0.081 |
|  | (0.095) |
| Household participated in Productive Safety Net Program (PSNP) | 0.012 |
|  | (0.108) |
| Log household consumption Per Adult Equivalent (PAE) per year | 0.125^**^ |
|  | (0.052) |
| Household owns a mobile phone | 1.385^***^ |
|  | (0.103) |
| Number of mobiles available in the household | 0.132^***^ |
|  | (0.046) |
| Household has access to electricity | 0.062 |
|  | (0.104) |
| Log (distance to nearest market, in KM) | -0.112^***^ |
|  | (0.041) |
| Log (distance to nearest town, in KM) | 0.008 |
|  | (0.042) |
| Region dummy: Afar | 0.777^***^ |
|  | (0.149) |
| Region dummy: Amhara | 1.253^***^ |
|  | (0.138) |
| Region dummy: Oromia | 1.840^***^ |
|  | (0.138) |
| Region dummy: Somali | -0.024 |
|  | (0.165) |
| Region dummy: Benishangul Gumuz | 1.443^***^ |
|  | (0.170) |
| Region dummy: SNNP | 0.615^***^ |
|  | (0.146) |
| Gambela | 0.429^***^ |
|  | (0.159) |
| Harar | 0.719^***^ |
|  | (0.158) |
| Addis Ababa | 0.581^***^ |
|  | (0.142) |
| Dire Dawa | 0.417^***^ |
|  | (0.153) |
| Constant | -4.032^***^ |
|  | (0.600) |
| Number of observations | 6664 |

Notes: this table reports coefficients from a logit regression. The base education is those below upper primary education. The base asset quintile is the first quintile, and the base region is Tigray. Standard errors in parentheses. ^*^ *p* < 0.10, ^**^ *p* < 0.05, ^***^ *p* < 0.01.

Table G: Descriptive statistics of the sample, by sample weights

|  | Unweighted  full sample  (1) | Unweighted  phone survey  (2) | Weighted  phone survey  (3) |
| --- | --- | --- | --- |
| Household head age in years | 42.19 | 40.99 | 42.34 |
| Household head is female | 0.32 | 0.30 | 0.32 |
| Education of household head: Lower primary | 0.56 | 0.39 | 0.56 |
| Upper primary | 0.16 | 0.18 | 0.16 |
| Secondary or higher | 0.28 | 0.42 | 0.29 |
| Household size | 4.36 | 4.09 | 4.37 |
| Adult household members size | 2.45 | 2.51 | 2.45 |
| Household head engaged in agriculture | 0.56 | 0.47 | 0.56 |
| Household head engaged in wage earning | 0.30 | 0.36 | 0.29 |
| Household head engaged in non-farm business | 0.18 | 0.22 | 0.19 |
| Asset quintiles: First quintile | 0.20 | 0.10 | 0.19 |
| Second quintile | 0.24 | 0.14 | 0.23 |
| Third quintile | 0.16 | 0.14 | 0.17 |
| Fourth quintile | 0.20 | 0.28 | 0.20 |
| Fifth quintile | 0.20 | 0.33 | 0.21 |
| Household located in rural area | 0.46 | 0.28 | 0.46 |
| Household participated in PSNP | 0.13 | 0.08 | 0.13 |
| Household consumption PAE per year | 21880.51 | 25982.92 | 22071.15 |
| Log household consumption PAE per year | 9.68 | 9.91 | 9.72 |
| Household owns a mobile phone | 0.67 | 0.90 | 0.67 |
| Number of mobiles available in the household | 1.14 | 1.66 | 1.16 |
| Household has access to electricity | 0.52 | 0.72 | 0.53 |
| Distance to market in KM | 54.98 | 37.66 | 52.69 |
| Distance to nearest town in KM | 26.75 | 18.59 | 25.78 |
| Log (distance to market in KM) | 3.23 | 2.75 | 3.21 |
| Log (distance to nearest town in KM) | 2.66 | 2.27 | 2.62 |
| Tigray | 0.10 | 0.06 | 0.09 |
| Afar | 0.08 | 0.06 | 0.07 |
| Amhara | 0.11 | 0.11 | 0.11 |
| Oromia | 0.11 | 0.15 | 0.12 |
| Somali | 0.09 | 0.04 | 0.13 |
| Benishangul Gumuz | 0.05 | 0.06 | 0.05 |
| SNNP | 0.10 | 0.06 | 0.09 |
| Gambella | 0.07 | 0.06 | 0.07 |
| Harar | 0.08 | 0.10 | 0.08 |
| Addis Ababa | 0.11 | 0.19 | 0.12 |
| Dire Dawa | 0.09 | 0.10 | 0.07 |
| Other highland regions | 0.32 | 0.32 | 0.32 |
| Other regions | 0.90 | 0.94 | 0.91 |
| Number of observations | 6770 | 2677 | 2677 |
